# Atrial Fibrillation, Neurocognitive Decline and Gene Expression After Cardiopulmonary Bypass

**DOI:** 10.5935/1678-9741.20150070

**Published:** 2015

**Authors:** Rahul S. Dalal, Ashraf A. Sabe, Nassrene Y. Elmadhun, Basel Ramlawi, Frank W. Sellke

**Affiliations:** 1 Division of Cardiothoracic Surgery, Cardiovascular Research Center, Warren Alpert Medical School of Brown University, Providence, RI, USA.; 2 Methodist DeBakey Heart & Vascular Center, Methodist Hospital, Houston, Texas, USA.

**Keywords:** Atrial Fibrillation, Cardiopulmonary Bypass, Genes, Microarray Analysis

## Abstract

**OBJECTIVE:**

Atrial fibrillation and neurocognitive decline are common complications after cardiopulmonary bypass. By utilizing genomic microarrays we investigate whether gene expression is associated with postoperative atrial fibrillation and neurocognitive decline.

**METHODS:**

Twenty one cardiac surgery patients were prospectively matched and underwent neurocognitive assessments pre-operatively and four days postoperatively. The whole blood collected in the pre-cardiopulmonary bypass, 6 hours after-cardiopulmonary bypass, and on the 4^th^ postoperative day was hybridized to Affymetrix Gene Chip U133 Plus 2.0 Microarrays. Gene expression in patients who developed postoperative atrial fibrillation and neurocognitive decline (n=6; POAF+NCD) was compared with gene expression in patients with postoperative atrial fibrillation and normal cognitive function (n=5; POAF+NORM) and patients with sinus rhythm and normal cognitive function (n=10; SR+NORM). Regulated genes were identified using JMP Genomics 4.0 with a false discovery rate of 0.05 and fold change of >1.5 or <-1.5.

**RESULTS:**

Eleven patients developed postoperative atrial fibrillation. Six of these also developed neurocognitive decline. Of the 12 patients with sinus rhythm, only 2 developed neurocognitive decline. POAF+NCD patients had unique regulation of 17 named genes preoperatively, 60 named genes six hours after cardiopulmonary bypass, and 34 named genes four days postoperatively (*P*<0.05) compared with normal patients. Pathway analysis demonstrated that these genes are involved in cell death, inflammation, cardiac remodeling and nervous system function.

**CONCLUSION:**

Patients who developed postoperative atrial fibrillation and neurocognitive decline after cardiopulmonary bypass may have differential genomic responses compared to normal patients and patients with only postoperative atrial fibrillation, suggesting common pathophysiology for these conditions. Further exploration of these genes may provide insight into the etiology and improvements of these morbid outcomes.

**Table:** 

**Abbreviations, acronyms & symbols**	
AF	= Atrial fibrillation
CABG	= Coronary artery bypass graft
CPB	= Cardiopulmonary bypass
NCD	= Neurocognitive decline
POAF	= Postoperative atrial fibrillation
SR	= Sinus rhythm

## INTRODUCTION

Surgical advancements have allowed an increasingly older population to undergo cardiac surgery and cardiopulmonary bypass (CPB) with a low mortality risk. Efforts have therefore focused on reducing postoperative morbidity. Neurocognitive decline (NCD, up to 80% incidence) and atrial fibrillation (AF, 20-45% incidence) remain two of the most common complications after CPB^[1,2]^. Coronary artery bypass graft (CABG) guidelines by the American College of Cardiology/American Heart Association describe two types of neurocognitive deficits, with type 2 representing the vast majority^[3]^. Type 2 deficits are global and may include confusion and intellectual and memory decline without a known focal lesion and may significantly impair patients' quality of life. The etiology of these deficits is likely related to multiple factors including age, procedure, CPB time, hypoxia, and inflammation^[4]^. Up to 30% of type 2 deficits persist for at least one year and early NCD appears to predict long-term deficits^[5]^.

Like NCD, the high incidence of postoperative AF (POAF) has persisted. POAF generally occurs by postoperative day four and may precipitate heart failure and cerebrovascular emboli^[6-8]^. Because of increased hospital stay and readmissions, it is estimated that healthcare costs for patients who develop POAF are $10,000 higher than for those who do not^[7]^. Though several factors have been correlated with POAF after cardiac surgery, our inability to eliminate its incidence may be related to unknown pathophysiologic mechanisms. Studies have proposed that oxidation and inflammation after CPB induce cardiomyocyte damage and predispose to the development of atrial arrhythmias^[9]^. Experiments in a canine model of rapid atrial pacing demonstrated that statins, which are known for their anti-inflammatory and anti-oxidant properties, reduced shortening of the atrial effective refractory period and thus POAF susceptibility^[10]^. In a case-control study, our group previously demonstrated that patients with POAF had elevated serum peroxide levels, excess myocardial oxidation, and an increased oxidative genomic response compared with patients in sinus rhythm (SR)^[11]^.

While these complications have been studied independently, prior research suggests an association between POAF and neurologic abnormalities^[12]^. In a prospective observational study, Stanley et al.^[13]^ found significantly more cognitive deficits in patients who developed POAF, which was also associated with worse cognitive functioning six weeks after surgery. While it is thought that the paroxysmal nature of POAF, embolization, and decreased cardiac output increase risk for neurologic dysfunction, it remains unknown if there are common pathways by which both NCD and POAF arise.

High-throughput microarray provides a practical approach to investigate genomic changes and disease development. Microarrays can screen the entire human genome for regulated genes and bring light to the underlying pathways that may promote morbidities like NCD and POAF. We previously utilized microarray to demonstrate increased expression of genes involved with inflammation and neurologic dysfunction in patients who developed NCD after CPB compared to patients without NCD (NORM)^[14]^. We now examine gene expression changes in patients who develop both POAF and NCD (POAF+NCD) compared to patients spared of these complications (SR+NORM) and those who develop POAF alone (POAF+NORM). To further investigate the underlying pathophysiology of these disease processes we utilize modern microarray and bioinformatics techniques to identify genes that may be associated with the combined incidence of these complications.

## METHODS

### Patient Enrollment and Matching

We performed a single-institution, prospective cohort study approved by the Beth Israel Deaconess Medical Center Institutional Review Board/Committee on Clinical Investigations in Boston, MA. Forty-two consecutive patients were scheduled for urgent or elective primary CABG, valve replacement (mitral or aortic), or a combination of both requiring CPB. All study participants were provided informed written consent for surgical procedures and blood collection for this investigation. Patients with pre-operative documented AF, high-grade carotid stenosis, known calcified aortas, recent cerebrovascular accident, severe neurologic deficits, serum creatinine>2.0 mg/dL, and hepatic cirrhosis were excluded. Subjects undergoing aortic root/arch procedures, on antiarrhythmic medications, or unable to complete neurocognitive assessments were also excluded.

POAF was defined as sustained AF confirmed by electrocardiogram before postoperative day five that required anticoagulation or cardioversion. Of the 42 subjects enrolled, only the subset that developed both POAF and NCD was prospectively matched with selected SR+NORM and POAF+NORM patients based on pre-operative baseline characteristics (i.e. sex, age, hypercholesterolemia, hypertension, diabetes mellitus, white blood cell count, β-blocker use), intraoperative characteristics (i.e. CPB and aortic cross-clamp time, cardiotomy suction and antifibrinolytic use, procedure type), and postoperative characteristics (i.e. β-blocker use and time to extubation). Subsequent serologic and molecular studies were performed in a blinded fashion.

### Surgical Technique

We followed our institution's conventional operative approach regarding general anesthesia induction, midline sternotomy, systemic heparinization, CPB, and invasive monitoring as previously described^[14]^.

### Neurocognitive Assessment

Patients underwent neurocognitive assessments performed by trained, blinded psychometricians between 1 and 10 days pre-operatively, on postoperative day 4, and in the 3^rd^ month of the postoperative period. Patients were also evaluated for depression using the Geriatric Depression Scale. Memory, attention, language, global cognition, and executive functioning were assessed using 8 validated tools:

The Hopkins Verbal Learning Test measured verbal learning, recall, and retention by assessing the maximum number of items learned, the number of items recalled after 20 minutes divided by the maximum number learned, and the number of items correctly named from a list. Working memory and attention span were measured using Digit Span. Attention shifting ability was assessed by recording the time needed to complete Trailmaking A and B. Confrontational naming was measured using the Boston Naming Test. Fluency was evaluated by requiring patients to generate words beginning with a specific letter (phonemic fluency) or in a category (semantic fluency). The Visual Search and Acuity Test and Stroop Color-Word Inference Test measured visuospatial abilities and executive function. Premorbid intelligence was measured using the Wechsler Test of Adult Reading. In accordance with the "Statement of consensus on assessment of neurobehavioral outcomes after cardiac surgery," NCD was defined as a 1-standard deviation deficit from baseline on 25% of tasks^[15]^.

### Blood Collection and Microarray Processing

Blood samples were drawn from patients via central venous catheter pre-operatively immediately after anesthesia induction (pre-CPB), 6 hours postoperatively in the intensive care unit (post-CPB), and on postoperative day four (4D). Whole blood was drawn into PAXgene tubes (QIAGEN Inc, Valencia, Ca) for extraction and mRNA stabilization per the manufacturer's instructions.

RNA extraction and purification from whole blood, cDNA synthesis, and generation of biotin-labeled cRNA were performed by the Beth Israel Deaconess Medical Center Proteomics Core according to prior protocols^[16,17]^. All cRNA samples were hybridized to Affymetrix GeneChip HG-U133 Plus 2.0 microarrays (Affymetric INc, Santa Clara, Ca). Chips were scanned using the HP G2500A ChipScanner (Affymetrix) and dChip software (Wong et al.^[18]^, Boston, MA) was used for quality control analysis and signal measurement. No outliers were identified and all samples underwent subsequent pathway analysis.

### Gene Expression and Pathway Analysis

Raw microarray data underwent gene expression analysis using JMP Genomics 4.0 (SAS, Cary, NC) for normalization, quality control, and statistical analysis. The Robust Multichip Average method normalized and compared composite chip data. Gene expression in Pre-CPB, Post-CPB, and 4D blood samples for POAF+NCD patients were compared to corresponding samples from SR+NORM and POAF+NORM using one-way ANOVA. A post-hoc false discovery rate algorithm with alpha of 0.05 minimized false positive results. Significantly, regulated genes met two criteria: 1) -log (*P*-value) exceeding the threshold calculated by JMP Genomics for each comparison and 2) fold change in gene expression >1.5 or <-1.5 between groups. A 1.5-fold change cutoff was chosen here and in a prior study of this patient population to reduce background noise while not limiting results to the most labile genes^[14,19]^. Significantly regulated genes were uploaded into Ingenuity Pathway Analysis (IPA, Ingenuity Systems, Redwood City, CA) to generate top canonical pathways regulated by the selected genes.

### Real-time PCR

Gene expression analysis of whole blood-derived mRNA with Affymetrix GeneChip HG-U133 Plus 2.0 microarrays was validated previously by real-time PCR^[20]^.

## RESULTS

### Patient Characteristics

Patients with POAF+NCD (n=6) were prospectively matched with SR+NORM (n=10) and POAF+NORM (n=5). Table 1 lists well-matched baseline characteristics of these subjects and shows no significant differences in race, sex, age, and co-morbidities as calculated by one-way ANOVA. Patients underwent similar intraoperative courses with regard to anesthesia, CPB technique, temperature, and perioperative monitoring. There were no differences in other postoperative complications, such as focal neurologic deficits or cerebrovascular events in patients with POAF compared to SR during the study period. Of 11 total POAF patients, 6 developed NCD (54.5%), and of 12 SR patients, only 2 developed NCD (16.7%). After three months, all but one patient returned regained normal cognitive function^[20]^.

**Table 1:** Characteristics for matching of patients who developed POAF and NCD with controls.

Characteristic	A POAF+NCD (n=6)	B SR+NORM (n=10)	C POAF+NORM (n=5)	*P*-value
Pre-operative data				
Age (y)^a^	66.5±7.4	69.2±7.1	73.4±5.8	0.28
Sex (% male)	83.3 (5/6)	100** (**10**/**10**)**	80.0 (4/5)	0.40
Hypertension (% of group)	83.3 (5/6)	70.0 (7/10)	40.0 (2/5)	0.34
Hypercholesterolemia (% of group)	50.0 (3/6)	50.0 (5/10)	20.0 (1/5)	0.54
Diabetes mellitus (% of group)	50.0 (3/6)	30.0 (3/10)	40.0 (2/5)	0.76
Leukocytes (103 cells/µL)^a^	7.4±2.1	7.2±2.0	10.3±2.9	0.05
Hematocrit (%)	35.6±4.3	34.5±4.0	37.7±7.6	0.53
Glucose (mg/dL)	193±131	163±68	118±38	0.38
Intraoperative data				
Procedure (% CABG)	83.3 (5/6)	70.0 (7/10)	80.0 (4/5)	0.84
CPB time (min)^a^	78.3±32.6	78.9±26.3	70.6±20.1	0.84
Cross-clamp time (min)^a^	57.7±23.9	63.0±21.0	46.4±21.3	0.40

a *Values are mean* ± *SD*

CABG=coronary artery bypass graft; CPB=cardiopulmonary bypass; POAF=post-operative atrial fibrillation; SR=sinus rhythm

### Gene Expression and Confirmation

We previously published comprehensive gene expression databases of patients with POAF or SR before and after CPB as well as patients with and without NCD after CPB, including unsupervised hierarchical sample clustering, and confirmation of microarray gene-expression data with real-time PCR^[11,20]^. Our described microarray GeneChip identified 54,675 transcripts. Complete lists of genes regulated in the comparisons of POAF+NCD vs. SR+NORM or POAF+NORM are provided in Tables 2 to 7.

**Table 2:** Pre-CPB gene expression in patients with POAF+NCD compared with SR+NORM - complete list.

Accession ID	Gene Name	FC	*P*-values
ADM2	adrenomedullin 2	1.66	1.12E-05
CA11	carbonic anhydrase XI	1.58	4.47E-05
CD101	CD101 molecule	2.19	1.15E-04
COMTD1	catechol-O-methyltransferase domain containing 1	1.81	2.29E-05
GAS6-AS1	GAS6 antisense RNA 1	1.54	5.37E-05
KCNIP3	Kv channel interacting protein 3, calsenilin	1.56	2.51E-22
MCF2L	MCF.2 cell line derived transforming sequence-like	1.52	1.00E-04
MECR	mitochondrial trans-2-enoyl-CoA reductase	1.52	2.19E-07
MMP11	matrix metallopeptidase 11 (stromelysin 3)	1.71	5.01E-15
NUTM2F/NUTM2G	NUT family member 2G	1.89	6.31E-13
PHF20	PHD finger protein 20	0.65	1.78E-05
PYCR1	pyrroline-5-carboxylate reductase 1	1.53	5.37E-05
RGS12	regulator of G-protein signaling 12	1.52	9.77E-06
TOM1L2	target of myb1-like 2 (chicken)	1.60	1.15E-04
VGLL1	vestigial like 1 (Drosophila)	1.87	3.02E-07
WIZ	widely interspaced zinc finger motifs	1.83	8.91E-10
ZBED5	zinc finger, BED-type containing 5	1.63	1.07E-04

FC=fold change

**Table 7:** 4D gene expression in patients with POAF+NCD compared with POAF+NORM - complete list.

Accession ID	Gene Name	FC	*P*-values
ACSL6	acyl-CoA synthetase long-chain family member 6	1.62	8.13E-06
ADAMTS6	ADAM metallopeptidase with thrombospondin type 1 motif, 6	0.65	4.37E-05
ADRBK2	adrenergic, beta, receptor kinase 2	0.22	1.29E-09
AGPAT6	1-acylglycerol-3-phosphate O-acyltransferase 6	1.63	1.58E-12
BCL2L1	BCL2-like 1	2.75	3.09E-06
C20orf203	chromosome 20 open reading frame 203	0.31	3.16E-14
CASC7	cancer susceptibility candidate 7 (non-protein coding)	1.75	1.05E-10
CBL	Cbl proto-oncogene, E3 ubiquitin protein ligase	0.64	3.02E-10
CDC42BPA	CDC42 binding protein kinase alpha (DMPK-like)	2.24	2.24E-10
CDCA7	cell division cycle associated 7	1.88	1.70E-09
CHD2	chromodomain helicase DNA binding protein 2	0.48	2.40E-05
CHERP	calcium homeostasis endoplasmic reticulum protein	0.46	8.91E-07
CLIC2	chloride intracellular channel 2	2.08	5.01E-27
DCAF15	DDB1 and CUL4 associated factor 15	0.48	5.89E-05
DDX17	DEAD (Asp-Glu-Ala-Asp) box helicase 17	6.55	2.29E-06
DLD	dihydrolipoamide dehydrogenase	1.89	2.51E-25
DOCK1	dedicator of cytokinesis 1	2.10	1.00E-10
EPB41L4B	erythrocyte membrane protein band 4.1 like 4B	0.65	7.94E-49
FRMD8	FERM domain containing 8	3.33	2.51E-17
GLCCI1	glucocorticoid induced transcript 1	2.43	1.29E-08
GRB10	growth factor receptor-bound protein 10	1.72	7.94E-14
HEMGN	hemogen	2.75	1.45E-09
IDE	insulin-degrading enzyme	1.56	2.82E-05
L1CAM	L1 cell adhesion molecule	1.60	1.05E-05
LOC100505812	uncharacterized LOC100505812	0.55	5.01E-11
MED1	mediator complex subunit 1	0.45	6.03E-06
MMD	monocyte to macrophage differentiation-associated	1.53	3.09E-09
MS4A6A	membrane-spanning 4-domains, subfamily A, member 6A	2.48	7.94E-14
NCR1	natural cytotoxicity triggering receptor 1	2.45	1.07E-04
NEDD4L	neural precursor cell expressed, developmentally down-regulated 4-like, E3 ubiquitin protein ligase	1.57	2.04E-07
ODF4	outer dense fiber of sperm tails 4	0.56	1.00E-09
OSBPL11	oxysterol binding protein-like 11	0.28	2.51E-26
PRDM2	PR domain containing 2, with ZNF domain	0.48	3.09E-05
PTAR1	protein prenyltransferase alpha subunit repeat containing 1	0.53	8.13E-06
PTPLB	protein tyrosine phosphatase-like (proline instead of catalytic arginine), member b	0.46	8.71E-05
PTPN9	protein tyrosine phosphatase, non-receptor type 9	0.61	4.68E-05
RAB32	RAB32, member RAS oncogene family	0.58	1.15E-04
RASSF1	Ras association (RalGDS/AF-6) domain family member 1	0.46	3.24E-06
RBM12B	RNA binding motif protein 12B	1.52	3.98E-11
REEP1	receptor accessory protein 1	1.72	2.45E-07
RPL10	ribosomal protein L10	0.36	7.08E-09
SERPINE1	serpin peptidase inhibitor, clade E (nexin, plasminogen activator inhibitor type 1), member 1	0.59	4.79E-05
SGOL1	shugoshin-like 1 (S. pombe)	0.56	2.69E-06
SLC22A7	solute carrier family 22 (organic anion transporter), member 7	0.54	7.94E-12
SLC5A4	solute carrier family 5 (low affinity glucose cotransporter), member 4	0.50	3.02E-05
SMC3	structural maintenance of chromosomes 3	1.72	3.16E-27
TCF4	transcription factor 4	0.63	1.38E-07
UBE2H	ubiquitin-conjugating enzyme E2H	2.64	1.58E-06
VPS37A	vacuolar protein sorting 37 homolog A (S. cerevisiae)	1.71	1.10E-04
WHAMMP2	WAS protein homolog associated with actin, golgi membranes and microtubules pseudogene 2	0.58	5.75E-08
YOD1	YOD1 deubiquitinase	1.53	3.47E-05
ZEB1	zinc finger E-box binding homeobox 1	0.56	1.26E-04
ZNF395	zinc finger protein 395	0.53	1.10E-06

FC=fold change

### Gene Expression and Pathway Analysis in POAF+NCD vs. SR+NORM

**Fig. 1:**
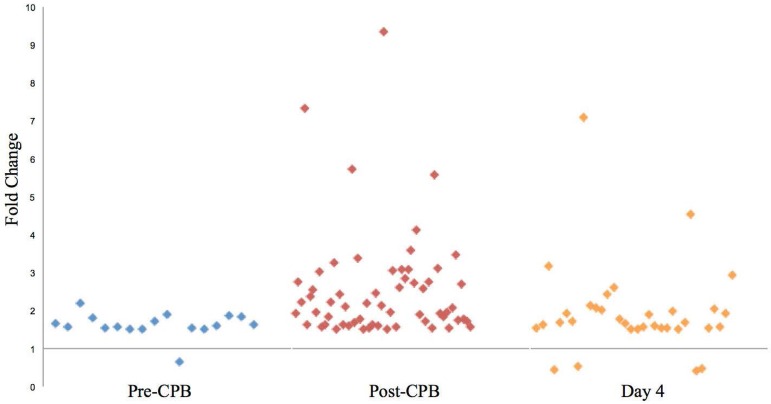
Distribution of genes regulated for POAF+NCD *vs.* SR+NORM.

Figure 1 shows the distribution of regulated genes by fold-change for each time point in this comparison. Pre-CPB, 19 genes were significantly regulated in the POAF+NCD group compared to NORM+SR, of which 17 were named. Notably, 16 of these 17 genes were up-regulated, while 1 was down-regulated. Pathway analysis used to group genes by potential pathophysiologic functions demonstrated that these genes are related to cardiovascular disease, nervous system function, and cell death, as described in Table 8. Post-CPB, the number of genes increased to 65, of which 60 were named. All 60 were up-regulated, and while distinct from those regulated pre-operatively, pathway analysis demonstrated that many of these genes are associated with cardiovascular disease and remodeling, inflammation, and nervous system disorders, as seen in Table 9. At 4D, the number of genes decreased to 41, of which 34 were named. Of these, 30 were up-regulated while 4 were down-regulated. Several genes, as listed in Table 10, are similarly involved with cardiovascular disease, nervous system function, inflammation, and protein degradation.

**Table 8:** Pre-CPB Gene Expression in Patients with POAF and NCD compared with SR and NORM - selected genes grouped by poten-tial pathophysiologic function.

Accession ID	Gene Name	FC	*P*-values
Cardiovascular disease			
ADM2	adrenomedullin-2	1.66	1.00E-04
Nervous system function			
KCNIP3	Kv channel interacting protein 3, calsenilin	1.56	2.51E-22
Cell death and survival			
MMP11	matrix metallopeptidase 11 (stromelysin 3)	1.71	5.01E-15

FC=fold change

**Table 9:** Post-CPB gene expression in patients with POAF and NCD compared with SR and NORM - selected genes grouped by poten-tial pathophysiologic function.

Accession ID	Gene Name	FC	*P*-values
Cardiovascular disease			
BMX	BMX non-receptor tyrosine kinase	7.32	1.00E-14
EPAS1	endothelial PAS domain protein 1	2.43	6.17E-05
HGF	hepatocyte growth factor (hepapoietin A; scatter factor)	1.79	3.47E-05
MAPK14	mitogen-activated protein kinase 14	1.95	2.51E-28
Nervous system function			
KIDINS220	kinase D-interacting substrate, 220kDa	1.54	7.08E-06
SYNE1	spectrin repeat containing, nuclear envelope 1	3.11	3.09E-06
YKT6	YKT6 v-SNARE homolog (S. cerevisiae)	1.72	3.39E-05
Inflammation			
CREBBP	CREB binding protein	1.83	1.29E-06
Pyschological disorders			
TMLHE	trimethyllysine hydroxylase, epsilon	1.95	3.47E-05

FC=fold change

**Table 10:** 4D Gene expression in patients with AF and NCD compared with SR and NORM – selected genes grouped by potential pathophysiologic function.

Accession ID	Gene Name	FC	*P*-values
Cardiovascular disease			
BCL2L1	BCL2-like 1	3.17	1.58E-13
PRKAA2	protein kinase, AMP-activated, alpha 2 catalytic subunit	1.54	5.37E-05
Nervous system function			
IDE	insulin-degrading enzyme	1.52	6.61E-06
CDC42BPA	CDC42 binding protein kinase alpha (DMPK-like)	1.94	1.86E-08
PLXNB1	plexin B1	1.53	1.00E-15
Inflammation			
NCR1	natural cytotoxicity triggering receptor 1	1.90	2.57E-05
DOCK1	dedicator of cytokinesis 1	2.09	6.31E-50
SMC3	structural maintenance of chromosomes 3	1.53	4.47E-06
Protein degradation			
DLD	dihydrolipoamide dehydrogenase	2.13	3.16E-14
NEDD4L	neural precursor cell expressed, developmentally down-regulated 4-like, E3 ubiquitin protein ligase	1.60	6.31E-11
UBE2H	ubiquitin-conjugating enzyme E2H	2.94	7.76E-06

FC=fold change

### Gene Expression and Pathway Analysis in Patients with POAF+NCD vs. POAF+NORM.

**Fig. 2:**
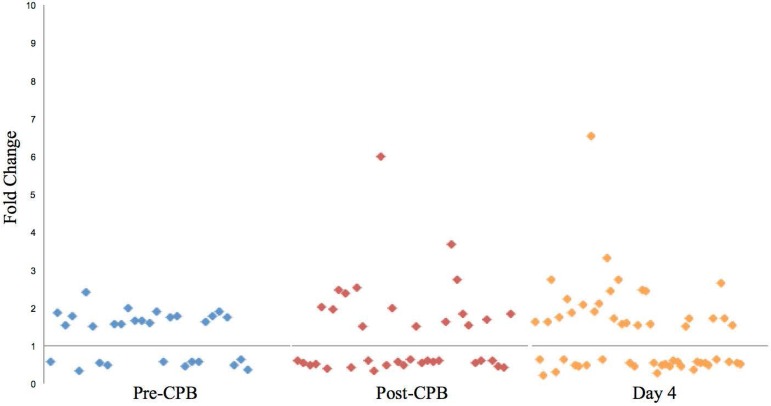
Distribution of genes regulated for POAF+NCD *vs.* POAF+NORM.

Figure 2 shows the distribution of regulated genes by fold-change for each time point. Pre-CPB 42 genes were significantly regulated in the POAF+NCD group compared to POAF+NORM, of which 29 were named. Of these, 18 were up-regulated, while 11 were down-regulated. These genes were associated with cardiovascular disease, nervous system function, and inflammation. Post-CPB, the number of regulated genes was 39, of which 37 were named. Sixteen of these 37 were up-regulated, while 21 were down-regulated. Pathway analysis demonstrated that these genes serve roles in cardiovascular disease and inflammation. At 4D, the number of regulated genes increased to 72, of which 54 were named. Twenty-seven of these were up-regulated, while 27 were down-regulated. IPA analysis again revealed that several genes affect cardiovascular disease, inflammation, and cell death. Selected genes grouped by pathophysiologic function for the POAF+NCD vs. POAF+NORM comparisons are found in Tables 11-13. While the majority of the genes identified for these comparisons were distinct from that of POAF+NCD vs. SR+NORM across all time points, multiple genes overlap and are listed in Table 14.

**Table 11:** Pre-CPB gene expression in patients with POAF+NCD compared with POAF+NORM - selected genes grouped by potential pathophysiologic function.

Accession ID	Gene Name	FC	*P*-values
Cardiovascular disease/function			
NFATC1	nuclear factor of activated T-cells, cytoplasmic, calcineurin-dependent 1	1.61	6.46E-05
TUBG1	tubulin, gamma 1	1.79	8.71E-05
MCF2L	MCF.2 cell line derived transforming sequence-like	1.66	4.17E-05
Nervous system function			
FKRP	fukutin related protein	0.54	4.17E-05
KCNIP3	Kv channel interacting protein 3, calsenilin	1.58	6.31E-19

FC=fold change

**Table 12:** Post-CPB gene expression in patients with POAF+NCD compared with POAF+NORM – selected genes grouped by potential pathophysiologic function.

Accession ID	Gene Name	FC	*P*-values
Cardiovascular disease/function			
MAPK14	mitogen-activated protein kinase 14	1.98	6.31E-24
SYNE1	spectrin repeat containing, nuclear envelope 1	2.74	1.26E-04
CDS2	CDP-diacylglycerol synthase (phosphatidate cytidylyltransferase) 2	2.03	3.09E-09
Inflammation			
HIVEP2	human immunodeficiency virus type I enhancer binding protein 2	0.62	6.03E-05

FC=fold change

**Table 13:** 4D Gene expression in patients with POAF+NCD compared with POAF+NORM - selected genes grouped by potential patho-physiologic function.

Accession ID	Gene Name	FC	*P*-values
Cardiovascular disease			
CBL	Cbl proto-oncogene, E3 ubiquitin protein ligase	0.64	2.24E-10
SERPINE1	serpin peptidase inhibitor, clade E (nexin, plasminogen activator inhibitor type 1), member 1	0.59	2.69E-06
BCL2L1	BCL2-like 1	2.75	3.09E-06
MED1	mediator complex subunit 1	0.45	3.09E-09
RASSF1	Ras association (RalGDS/AF-6) domain family member 1	0.46	3.98E-11
Cell death/survival			
IDE	insulin-degrading enzyme	1.56	1.05E-05
RAB32	RAB32, member RAS oncogene family	0.58	3.24E-06
CDC42BPA	CDC42 binding protein kinase alpha (DMPK-like)	2.24	1.70E-09
DOCK1	dedicator of cytokinesis 1	2.10	7.94E-49
L1CAM	L1 cell adhesion molecule	1.60	5.01E-11
PTPN9	protein tyrosine phosphatase, non-receptor type 9	0.61	1.15E-04
SMC3	structural maintenance of chromosomes 3	1.72	1.38E-07
DDX17	DEAD (Asp-Glu-Ala-Asp) box helicase 17	6.55	2.51E-25
GRB10	growth factor receptor-bound protein 10	1.72	1.45E-09
PRDM2	PR domain containing 2, with ZNF domain	0.48	8.13E-06
TCF4	transcription factor 4	0.63	1.58E-06
ZEB1	zinc finger E-box binding homeobox 1	0.56	1.10E-06
Inflammation			
ADRBK2	adrenergic, beta, receptor kinase 2	0.22	1.29E-09
NCR1	natural cytotoxicity triggering receptor 1	2.45	2.04E-07

FC=fold change

**Table 14:** Significantly regulated genes overlapping across multiple comparisons.

Comparisons	Overlapping Regulated Genes
POAF+NCD *vs.* SR+NORM (Pre-CPB)	ca11, kcnip3, mcf2l, mmp11, nutm2f/nutm2g, pycr1, vgll1, wiz
POAF+NCD *vs.* AF+NORM (Pre-CPB)	
POAF+NCD *vs.* SR+NORM (Post-CPB)	cds2, clec2b, dach1, fkbp9, gtf2h2, hist2h2be, mapk14, slc39a8, sult1b1, syne1, timm23, tor1aip2, yipf4, znf350
POAF+NCD *vs.* AF+NORM (Post-CPB)	
POAF+NCD *vs.* SR+NORM (4D)	agpat6, bcl2l1, c20orf203, casc7, cdc42bpa, cdca7, ddx17, dld, dock1, frmd8, glcci1, grb10, ide, mmd, ncr1, nedd4l, reep1, rpl10, smc3, ube2h
POAF+NCD *vs.* AF+NORM (4D)	
POAF+NCD *vs.* SR+NORM (Post-CPB)	UBE2H
POAF+NCD *vs.* SR+NORM (4D)	
POAF+NCD *vs.* AF+NORM (4D)	

## DISCUSSION

AF and NCD after cardiac surgery have each been extensively studied. Much of the literature for POAF has pointed to inflammation and oxidative stress as promoting factors. Indeed, prior work from our group demonstrated significantly elevated genomic markers of oxidative stress in the blood of patients who develop POAF after CPB^[11]^. We similarly used microarray to study NCD patients and found increased expression of blood inflammatory mediators from those undergoing CPB^[14]^. Given that the genomic regulation of systemic cytotoxic insults such as oxidation and inflammation appear to promote POAF and NCD when studied individually, we sought to determine if genomic responses differ in patients who develop both complications.

Our current microarray study shows that the expression profiles of patients who develop both POAF and NCD after CPB differ from those who develop neither complication nor POAF alone. The greatest amount of gene regulation occurred postoperatively, suggesting that CPB may induce a differential genomic response in susceptible patients. Furthermore, POAF+NCD vs. POAF+NORM had the most gene regulation at 4D, while POAF+NCD vs. SR+NORM had the most gene regulation post-CPB with a largely different set of genes identified. This suggests that POAF and NCD after CPB may be linked pathophysiologically through mechanisms distinct from those inducing POAF alone, with more genomic changes occurring at an earlier stage.

Many genes regulated post-CPB in POAF+NCD vs. SR+NORM are associated with pathologic cardiac remodeling. One such gene includes BMX, a non-receptor tyrosine kinase. Mitchell-Jordan et al.^[21]^ demonstrated that BMX-knockout mice were resistant to massive cardiac hypertrophy following transverse aortic constriction relative to wild type, indicating a significant role for BMX in cardiac remodeling. If the impressive 7.32-fold up-regulation of BMX in the blood of our POAF+NCD patients also reflects their myocardial expression, excess cardiac remodeling after CPB may be a predisposing factor for POAF and NCD. Additional up-regulated genes identified in this group with reported roles in cardiac remodeling include EPAS1, HGF, and MAPK14^[22-24]^. While there is much evidence for oxidative stress in cardiac remodeling and AF^[25]^, our study found genes implicated in remodeling but not oxidative stress, perhaps due to our limited sample size. However, while Ramlawi et al.^[20]^ demonstrated genomic regulation of oxidative stress in POAF patients, they did not report genes directly related to cardiac remodeling. This difference may lie in the fact that our patients developed NCD in addition to POAF, introducing a potential association of cardiac remodeling with secondary neurologic effects.

Several genes identified in the POAF+NCD vs. SR+NORM comparison are also directly implicated in neurologic dysfunction. KIDINS220 was up-regulated post-CPB and has been shown to accumulate with tau protein in the brains of Alzheimer Disease patients^[26]^. At 4D, there was also increased expression of PLXNB1, which controls the behavior of microtubule tips and dendrite morphology^[27]^. Given its critical role in regulating the cytoskeleton and dendrite growth, it is postulated to be involved in the pathogenesis of several neurological disorders.

Genes related to inflammation and cell death were also identified in POAF+NCD vs. SR+NORM. KIDINS200, discussed above, has a known role in T-cell receptor-mediated T-cell activation in addition to its neurologic functions^[28]^. At 4D, up-regulated pro-inflammatory genes include NCR1 and DOCK. NCR1 encodes a natural killer cell receptor that triggers cytotoxicity, while DOCK1 is involved in cytoskeletal rearrangements required for phagocytosis^[29,30]^ Genes involved with protein degradation were also identified at 4D, including NEDD4L and UBE2H. NEDD4L encodes an E3 ubiquitin ligase and UBEH2 encodes ubiquitin-conjugating enzyme E2H, both of which target proteins for lysosomal degradation^[31,32]^. These genes have no established relationship to either POAF or NCD after CPB, but given that systemic inflammatory and catabolic processes are known contributors to both complications, the regulation of these proteins at the genomic level may be relevant^[33-36]^.

Our study has limitations, the most significant of which is the size of our patient population. A larger study may allow for the identification of more genes that may characterize complete pathways, such as the oxidative stress response, as opposed to our identification of several isolated genes related to various pathways. While our patients were well matched, our sample size also precludes us from respecting Hardy-Weinberg Equilibrium. However, we hope that our findings stimulate interest in larger studies of this nature.

Another limitation is our profiling gene expression in blood rather than heart or brain tissues, both of which were not feasible in this study and would not be a practical option for future patient management strategies. It is unknown if the genes involved with cardiovascular and neurologic function identified in blood reflect pathways in the heart and brain. However, several genes we identified may have systemic effects through inflammation and cell death that may secondarily damage both heart and brain tissue and predispose these individuals to POAF and NCD.

## CONCLUSION

Our findings may expand what is known about the pathophysiology underlying POAF and NCD. While we cannot assert a true genetic association between POAF and NCD given our limited sample size, our results suggest that differential genomic responses existed in our study sample of patients who developed both complications after cardiac surgery. There may have been an influence of pathologic cardiac remodeling and involvement of genes with known roles in inflammation, cell death, and nervous system function that may have promoted POAF and NCD in our patient population. We hope that the database of regulated genes provided by this work sparks further study of differentially expressed pathways that may deepen our understanding of these important and costly complications and potentially offer means of risk stratification and improved patient management.

**Table:** 

**Authors' roles & responsibilities**	
RSD	Analysis or interpretation of data; statistical analysis; final approval of the manuscript; study design
AAS	Study design; final approval of the manuscript
NYE	Study design; final approval of the manuscript
BR	Study design; final approval of the manuscript
FWS	Final approval of the manuscript; study design; implementation of projects/experiments

## Figures and Tables

**Table 3:** Post-CBP gene expression in patients with POAF+NCD compared with SR+NORM - complete list.

Accession ID	Gene Name	FC	*P*-values
ABHD13	abhydrolase domain containing 13	1.92	1.00E-05
ACOX1	acyl-CoA oxidase 1, palmitoyl	2.75	1.23E-04
ARPC1A	actin related protein 2/3 complex, subunit 1A, 41kDa	2.23	7.41E-05
BMX	BMX non-receptor tyrosine kinase	7.32	1.00E-14
C1GALT1C1	C1GALT1-specific chaperone 1	1.63	1.82E-08
C2orf76	chromosome 2 open reading frame 76	2.38	5.25E-08
C5orf30	chromosome 5 open reading frame 30	2.56	6.17E-06
CDS2	CDP-diacylglycerol synthase (phosphatidate cytidylyltransferase) 2	1.95	4.07E-10
CEACAM21	carcinoembryonic antigen-related cell adhesion molecule 21	3.02	8.71E-05
CLEC12A	C-type lectin domain family 12, member A	1.57	2.09E-05
CLEC2B	C-type lectin domain family 2, member B	1.62	1.95E-05
CREBBP	CREB binding protein	1.83	1.29E-06
DAB2	Dab, mitogen-responsive phosphoprotein, homolog 2 (Drosophila)	2.23	8.51E-05
DACH1	dachshund homolog 1 (Drosophila)	3.27	5.01E-11
DNAJC5	DnaJ (Hsp40) homolog, subfamily C, member 5	1.51	8.32E-07
EPAS1	endothelial PAS domain protein 1	2.43	6.17E-05
FAM114A2	family with sequence similarity 114, member A2	1.64	2.04E-06
FAM200B	family with sequence similarity 200, member B	2.12	1.86E-05
FBXO28	F-box protein 28	1.59	9.33E-05
FKBP9	FK506 binding protein 9, 63 kDa	5.72	1.58E-12
GNG2	guanine nucleotide binding protein (G protein), gamma 2	1.69	6.03E-05
GTF2H2	general transcription factor IIH, polypeptide 2, 44kDa	3.37	2.09E-10
HGF	hepatocyte growth factor (hepapoietin A; scatter factor)	1.79	3.47E-05
HIST2H2BE (includes others)	histone cluster 2, H2be	1.52	5.01E-28
HOOK3	hook homolog 3 (Drosophila)	2.19	1.12E-04
KIDINS220	kinase D-interacting substrate, 220kDa	1.54	7.08E-06
KLHL7	kelch-like family member 7	1.63	1.17E-04
KPNA1	karyopherin alpha 1 (importin alpha 5)	2.45	1.78E-05
LEMD2	LEM domain containing 2	1.59	1.66E-05
LOC100506229	uncharacterized LOC100506229	2.15	3.09E-05
LOC100506328	uncharacterized LOC100506328	9.36	2.51E-11
LOC285835	uncharacterized LOC285835	1.51	6.31E-05
MAPK14	mitogen-activated protein kinase 14	1.95	2.51E-28
MARCH5	membrane-associated ring finger (C3HC4) 5	3.07	5.50E-06
MLLT4	myeloid/lymphoid or mixed-lineage leukemia (trithorax homolog, Drosophila); translocated to, 4	1.57	9.77E-05
MTIF3	mitochondrial translational initiation factor 3	2.60	4.07E-05
NKAP	NFKB activating protein	3.08	3.63E-05
OXSR1	oxidative stress responsive 1	2.84	1.62E-08
PDSS1	prenyl (decaprenyl) diphosphate synthase, subunit 1	3.08	1.45E-05
PFKFB2	6-phosphofructo-2-kinase/fructose-2,6-biphosphatase 2	3.60	2.51E-06
PRKAG1	protein kinase, AMP-activated, gamma 1 non-catalytic subunit	2.73	5.13E-05
RASGEF1A	RasGEF domain family, member 1A	4.12	1.78E-05
RILPL1	Rab interacting lysosomal protein-like 1	1.89	6.03E-06
SFXN1	sideroflexin 1	2.59	1.02E-04
SLC39A8	solute carrier family 39 (zinc transporter), member 8	1.73	3.98E-13
ST6GALNAC3	ST6 (alpha-N-acetyl-neuraminyl-2,3-beta-galactosyl-1,3)-N-acetylgalac-tosaminide alpha-2,6-sialyltransferase 3	2.77	5.50E-06
STAG1	stromal antigen 1	1.54	2.00E-16
SULT1B1	sulfotransferase family, cytosolic, 1B, member 1	5.57	3.47E-10
SYNE1	spectrin repeat containing, nuclear envelope 1	3.11	3.09E-06
TANK	TRAF family member-associated NFKB activator	1.94	3.24E-05
TIMM23	translocase of inner mitochondrial membrane 23 homolog (yeast)	1.84	2.51E-16
TMLHE	trimethyllysine hydroxylase, epsilon	1.95	3.47E-05
TOR1AIP2	torsin A interacting protein 2	1.55	6.31E-25
TRPS1	trichorhinophalangeal syndrome I	2.09	7.08E-05
UBE2H	ubiquitin-conjugating enzyme E2H	3.47	2.19E-07
VAMP3	vesicle-associated membrane protein 3	1.74	8.51E-05
WDFY3	WD repeat and FYVE domain containing 3	2.69	1.02E-05
YIPF4	Yip1 domain family, member 4	1.79	1.23E-06
YKT6	YKT6 v-SNARE homolog (S. cerevisiae)	1.72	3.39E-05
ZNF350	zinc finger protein 350	1.57	5.13E-05

FC=fold change

**Table 4:** 4D gene expression in patients with POAF+NCD compared with SR+NORM - complete list.

Accession ID	Gene Name	FC	*P*-values
AGPAT6	1-acylglycerol-3-phosphate O-acyltransferase 6	1.54	5.61E-04
ATP13A4	ATPase type 13A4	1.63	6.46E-06
BCL2L1	BCL2-like 1	3.17	1.58E-13
C20orf203	chromosome 20 open reading frame 203	0.45	4.37E-07
CASC7	cancer susceptibility candidate 7 (non-protein coding)	1.70	3.98E-11
CDC42BPA	CDC42 binding protein kinase alpha (DMPK-like)	1.94	1.86E-08
CDCA7	cell division cycle associated 7	1.72	5.75E-05
CTSO	cathepsin O	0.54	8.71E-05
DDX17	DEAD (Asp-Glu-Ala-Asp) box helicase 17	7.10	5.01E-27
DLD	dihydrolipoamide dehydrogenase	2.13	3.16E-14
DOCK1	dedicator of cytokinesis 1	2.09	6.31E-50
DSC2	desmocollin 2	2.01	4.07E-06
FRMD8	FERM domain containing 8	2.42	2.45E-06
GLCCI1	glucocorticoid induced transcript 1	2.61	3.16E-16
GRB10	growth factor receptor-bound protein 10	1.79	3.16E-11
HNMT	histamine N-methyltransferase	1.65	1.05E-04
IDE	insulin-degrading enzyme	1.52	6.61E-06
LOC284080	uncharacterized LOC284080	1.51	8.13E-05
MMD	monocyte to macrophage differentiation-associated	1.57	2.00E-15
NCR1	natural cytotoxicity triggering receptor 1	1.90	2.57E-05
NEDD4L	neural precursor cell expressed, developmentally down-regulated 4-like, E3 ubiquitin protein ligase	1.60	6.31E-11
PLXNB1	plexin B1	1.53	1.00E-15
PRKAA2	protein kinase, AMP-activated, alpha 2 catalytic subunit	1.54	5.37E-05
PRRT1	proline-rich transmembrane protein 1	1.98	3.98E-05
REEP1	receptor accessory protein 1	1.52	2.63E-07
RHCE/RHD	Rh blood group, D antigen	1.69	8.13E-05
RIOK3	RIO kinase 3	4.54	9.55E-05
RPL10	ribosomal protein L10	0.40	8.91E-05
RPL18	ribosomal protein L18	0.47	1.95E-05
SMC3	structural maintenance of chromosomes 3	1.53	4.47E-06
SRSF1	serine/arginine-rich splicing factor 1	2.04	4.37E-05
ST7	suppression of tumorigenicity 7	1.58	6.76E-05
TFAP2E	transcription factor AP-2 epsilon (activating enhancer binding protein 2 epsilon)	1.93	5.89E-06
UBE2H	ubiquitin-conjugating enzyme E2H	2.94	7.76E-06

FC=fold change

**Table 5:** Pre-CPB gene expression in patients with POAF+NCD compared with POAF+NORM - complete list.

Accession ID	Gene Name	FC	*P*-values
ACTR3BP5	ARP3 actin-related protein 3 homolog B (yeast) pseudogene 5	0.57	3.55E-09
AP5S1	adaptor-related protein complex 5, sigma 1 subunit	1.87	1.00E-04
C14orf166B	chromosome 14 open reading frame 166B	1.54	1.58E-12
CA11	carbonic anhydrase XI	1.79	8.91E-06
CCDC36	coiled-coil domain containing 36	0.35	5.01E-21
CIZ1	CDKN1A interacting zinc finger protein 1	2.42	1.10E-04
FHAD1	forkhead-associated (FHA) phosphopeptide binding domain 1	1.51	6.31E-06
FKRP	fukutin related protein	0.54	4.17E-05
GTPBP3	GTP binding protein 3 (mitochondrial)	0.49	2.75E-05
KCNIP3	Kv channel interacting protein 3, calsenilin	1.58	6.31E-19
KHSRP	KH-type splicing regulatory protein	1.56	4.79E-10
LOC100507477	uncharacterized LOC100507477	1.99	3.98E-05
MCF2L	MCF.2 cell line derived transforming sequence-like	1.66	4.17E-05
MMP11	matrix metallopeptidase 11 (stromelysin 3)	1.67	5.01E-12
NFATC1	nuclear factor of activated T-cells, cytoplasmic, calcineurin-dependent 1	1.61	6.46E-05
NUTM2F/NUTM2G	NUT family member 2G	1.89	3.16E-11
PHAX	phosphorylated adaptor for RNA export	0.59	2.45E-05
PYCR1	pyrroline-5-carboxylate reductase 1	1.75	5.89E-06
RIN1	Ras and Rab interactor 1	1.78	7.94E-05
SLC24A6	solute carrier family 24 (sodium/lithium/calcium exchanger), member 6	0.45	1.20E-04
SYT17	synaptotagmin XVII	0.57	3.98E-14
TACC2	transforming, acidic coiled-coil containing protein 2	0.57	3.16E-18
TMEM259	transmembrane protein 259	1.64	7.41E-06
TUBG1	tubulin, gamma 1	1.79	8.71E-05
VGLL1	vestigial like 1 (Drosophila)	1.90	1.74E-06
WIZ	widely interspaced zinc finger motifs	1.74	1.48E-07
WNK2	WNK lysine deficient protein kinase 2	0.48	3.98E-31
XYLT2	xylosyltransferase II	0.65	1.05E-04
ZNF528	zinc finger protein 528	0.36	8.51E-05

FC=fold change

**Table 6:** Post-CPB gene expression in patients with POAF+NCD compared with POAF+NORM - complete list.

Accession ID	Gene Name	FC	*P*-values
ANKMY2	ankyrin repeat and MYND domain containing 2	0.61	1.58E-12
ANKRD6	ankyrin repeat domain 6	0.54	1.38E-08
AP4E1	adaptor-related protein complex 4, epsilon 1 subunit	0.50	4.68E-05
BCS1L	BC1 (ubiquinol-cytochrome c reductase) synthesis-like	0.53	7.76E-05
CDS2	CDP-diacylglycerol synthase (phosphatidate cytidylyltransferase) 2	2.03	3.09E-09
CEBPG	CCAAT/enhancer binding protein (C/EBP), gamma	0.41	1.26E-05
CLEC2B	C-type lectin domain family 2, member B	1.97	1.70E-06
DACH1	dachshund homolog 1 (Drosophila)	2.47	8.32E-07
FKBP9	FK506 binding protein 9, 63 kDa	2.38	8.32E-05
GOLT1B	golgi transport 1B	0.42	3.98E-13
GTF2H2	general transcription factor IIH, polypeptide 2, 44kDa	2.54	1.35E-06
HIST2H2BE (includes others)	histone cluster 2, H2be	1.52	3.98E-26
HIVEP2	human immunodeficiency virus type I enhancer binding protein 2	0.62	6.03E-05
KMO	kynurenine 3-monooxygenase (kynurenine 3-hydroxylase)	0.33	1.00E-31
LOC100506328	uncharacterized LOC100506328	5.99	1.38E-07
LOC728613	programmed cell death 6 pseudogene	0.48	6.92E-05
MAPK14	mitogen-activated protein kinase 14	1.98	6.31E-24
NEK6	NIMA-related kinase 6	0.59	1.58E-21
PDS5B	PDS5, regulator of cohesion maintenance, homolog B (S. cerevisiae)	0.48	4.79E-05
PMM1	phosphomannomutase 1	0.63	1.66E-05
PPP2R1B	protein phosphatase 2, regulatory subunit A, beta	1.50	1.10E-05
RIOK1	RIO kinase 1	0.54	1.00E-15
RNF144B	ring finger protein 144B	0.60	2.88E-05
SIVA1	SIVA1, apoptosis-inducing factor	0.58	8.71E-08
SLC27A3	solute carrier family 27 (fatty acid transporter), member 3	0.60	1.58E-05
SLC39A8	solute carrier family 39 (zinc transporter), member 8	1.64	2.95E-10
SULT1B1	sulfotransferase family, cytosolic, 1B, member 1	3.68	3.09E-06
SYNE1	spectrin repeat containing, nuclear envelope 1	2.74	1.26E-04
TIMM23	translocase of inner mitochondrial membrane 23 homolog (yeast)	1.83	1.26E-13
TOR1AIP2	torsin A interacting protein 2	1.55	3.98E-22
TTLL12	tubulin tyrosine ligase-like family, member 12	0.54	3.55E-05
TUBB6	tubulin, beta 6 class V	0.61	8.13E-06
YIPF4	Yip1 domain family, member 4	1.68	8.32E-05
ZCCHC10	zinc finger, CCHC domain containing 10	0.61	2.09E-09
ZDHHC12	zinc finger, DHHC-type containing 12	0.47	4.37E-05
ZNF226	zinc finger protein 226	0.42	5.01E-29
ZNF350	zinc finger protein 350	1.83	7.94E-06

FC=fold change
